# Postoperative subphenotypes modified the hepatoma arterial-embolization prognostic score: A novel smHAP-II nomogram

**DOI:** 10.7150/jca.91175

**Published:** 2024-03-25

**Authors:** Linbin Lu, Wanting Guo, Jialin Chen, Simiao Gao, Lifang Liu, Baocuo Gong, Hongyi Yang, Xuewen Wang, Yayin Chen, Yanhong Shi, Xiong Chen

**Affiliations:** 1 Department of Oncology, Mengchao Hepatobiliary Hospital of Fujian Medical University, 350025, Fuzhou, Fujian, PR China.; 2 Department of Oncology, the 900th Hospital of Joint Logistic Support Force, PLA, Fuzong Clinical College of Fujian Medical University, 350025, Fuzhou, Fujian, PR China.; 3 Department of Oncology, the 900th Hospital of Joint Logistic Support Force, PLA, Xiamen University Medical College, 350025, Fuzhou, Fujian, PR China.

**Keywords:** Hepatocellular carcinoma, Trans-arterial chemo-embolization, prognosis, survival, smHAP-Ⅱ

## Abstract

**Background**: Three subphenotypes were identified for unresectable hepatocellular carcinoma (uHCC) after frontline transarterial chemoembolization (TACE). This study aimed to develop an individual smHAP-Ⅱ nomogram for uHCC patients after TACE.

**Methods**: Between January 2007 to December 2016, 1517 uHCC patients undergoing TACE were included from four hospitals in China (derivation cohort: 597 cases; validation cohort: 920 cases). Multivariable Cox proportion regression analysis was used to develop a nomogram, incorporating postoperative subphenotypes (Phenotype 1, 2, 3) and HAP score (Score 0 to 4). The model was validated by a 1000-time bootstrap resampling procedure. The performance of the model was compared with existing ones by Harrell's C-index and Area Under Curve (AUC).

**Results:** Postoperative subphenotypes modified the HAP score (smHAP-Ⅱ nomogram) was developed and validated, with the Harrell's C-index of the nomogram was 0.679 (SD: 0.029) for the derivation cohort and 0.727(SD:0.029) for the external cohort. The area under curves of the nomogram for 1-, 3-, and 5-year OS were 0.750, 0.710, and 0.732 for the derivation cohort, respectively (0.789, 0.762, and 0.715 for the external cohort). In the calibration curves stratified by treatment after TACE, the lines for re-TACE and stop-TACE cross at 0.23, indicating that patients with a 3-year predicted survival >23% would not benefit from TACE.

**Conclusions**: The addition of postoperative subphenotypes significantly improved the prognostic performance. The smHAP-Ⅱ nomogram can be used for accurate prognostication and selection of optimal candidates for TACE, with the value to guide sequential treatment strategy.

## 1. Introduction

Hepatocellular carcinoma (HCC), one of the most prevalent malignancies, leads to the majority of cancer deaths, particularly in China 1-3. Although progress has been made in the treatment, the prognosis remains very poor as a small group of patients with early-stage HCC has access to curative options such as liver transplantation, surgical resection and radiofrequency ablation 4. Transarterial chemoembolization (TACE) has long been recommended as the first-line treatment for the intermediate stage of HCC (BCLC stage B) 4, 5. There are, however, some patients who do not respond to TACE or show tumor progression in response to repeated sessions; thus, the optimal time for other potential treatments may be missed. The repeated use of TACE often leads to treatment-related complications 6, which may reduce the survival benefit of TACE. Furthermore, alternative treatment options are limited for the patients who received TACE as the first line 7-10. Despite many new target drugs and immunotherapies having shown efficacy as first- and second-line treatments for advanced HCC, the survival benefit is modest with high cost 11-14. There are no guidelines concerning the standard switch to another treatment strategy after initial treatment for intermediate-stage HCC.

In addition, patients with BCLC stage B HCC who have received TACE have a highly heterogeneous outcome prediction due to this population's enormous heterogeneity with a variable median overall survival of 13-43 months 15-18. Various risk prediction models have therefore been developed to predict treatment outcomes after TACE 19-27. A baseline tumor characteristic and liver function test were associated with HCC survival after TACE in these studies 24, 28, 29. For the first time, L. Kadalayil1 et al. 20 proposed a simple scoring system of intermediate HCC called the Hepatoma Arterial-embolization Prognostic (HAP) score, which has been specifically designed for predicting treatment outcomes, and a variety of independent cohorts have shown that accuracy still can be improved 24, 29. With the development of the HAP score, the modified HAP-II score (mHAP-II) offers the advantage of the ease of use and simplicity, but individual prognostications cannot be made 24. To overcome the shortcoming, the modified version of HAP-III (mHAP-III) includes both HAP variables and tumor number in their continuous (as opposed to dichotomized) form 30. On the other hand, it has been confirmed that the HAP score is HCC-specific rather than TACE-specific, and the modified models based on TACE response seem to stratify survival better 31.

Recently, we have identified 3-class postoperative subphenotypes in intermediate-stage HCC after first TACE using latent class models, and it is an independent risk factor for clinical outcome 32. Whether the addition of 3-class subphenotypes to HAP score will significantly improve the prognostic performance is still unclear. This study aims to develop an individual smHAP-Ⅱ nomogram for uHCC patients after TACE.

## 2. Methods and patients

### 2.1 Patients and study design

**Figure 1:**
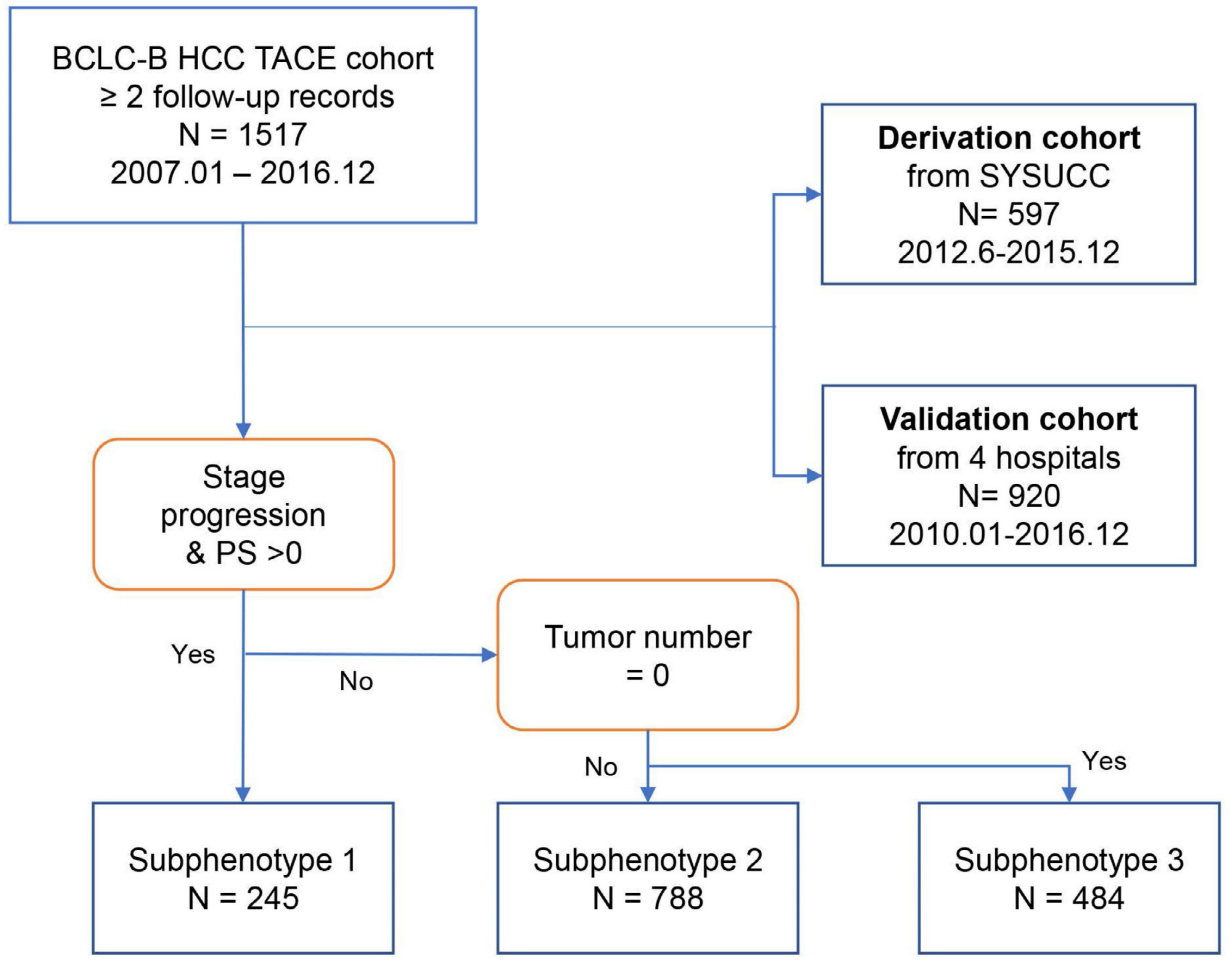
** Flow chart of patient analysis by phenotype.** Shown is the procedure of patients into each subphenotype with the indicated key variables: PS score, tumor number, and stage progress.

Data on 2020 patients who were diagnosed to have intermediate-stage HCC and underwent TACE from January 2007 to December 2016 were retrospectively reviewed 33-35. In this study, we chose the HCC patients with at the two follow-up records after TACE. The selection process according to inclusion and exclusion criteria have been published and shown in Figure 1 32. A total of 597 patients were included in the derivation cohort after excluding those who refused treatment (n=37, 3.8%), underwent surgery as first-line therapy (n=225, 23.0%), and had only one follow-up record (n=120, 12.3%). Additionally, analyses were replicated in an independent cohort (n=920), with 65 patients excluded from the internal testing cohort and 55 patients excluded from the multicenter testing cohort due to having only one follow-up record.

This study complied with the TRIPOD Statement 36 and obtained approval from The Department of Clinical Research of Sun Yat-sen University Cancer Center (2017-FXY-129) and The Hospital Ethics Committee of the four medical centers. Because this was a secondary analysis study, and the data were anonymous, the requirement for informed consent was waived. Patients or the public were not involved in the design, conduct, reporting, or dissemination plans of our research.

### 2.2 Definition of the postoperative subphenotypes and HAP score

As was shown in Figure 1, postoperative subphenotypes prediction 32 was based on three variables after first TACE, including PS score (0/1), No. intrahepatic lesions (0/≤3/>3), new intrahepatic lesion (no/yes), stage progress (no/yes). There were 3-class postoperative subphenotypes, including TACE-refractory subphenotype (Phenotype 1: PS score 1, stage progress, and more intrahepatic lesions), TACE-responsive subphenotype (Phenotype 3: PS score 0, No intrahepatic lesions), compared to TACE-intermediate subphenotype (Phenotype 2).

The HAP scoring system was based on the four predictors: albumin, bilirubin, AFP, and tumor size. Patients were assigned one point for each of the four parameters when they were in the adverse group as defined by the cut-off. The HAP score was defined as the sum of these scores, and patients were classified into low- (HAP A), intermediate-(HAP B), high-(HAP C), or very high-(HAP D) risk groups with HAP scores of 0, 1, 2 or >2 points, respectively 20.

### 2.3 Statistical analysis

OS was calculated from the date of the first session of TACE to the date of death or last follow-up. Quantitative data were expressed as frequency, mean ± standard deviation, or median with a 95% confidence interval (CI); Categorical variables were described as frequencies and percentages. Mann-Whitney U test was applied to compare continuous variables, and either Pearson's χ^2^ test or Fisher's exact test was performed for comparing categorical data. Cumulative survival curves of OS were estimated using the Kaplan-Meier method.

A prognostic nomogram was constructed using two independent predictors (postoperative subphenotypes, HAP score) assessed by the multivariate Cox proportional hazards model. The concordance index (C-index) was calculated to assess the performance of the nomograms. Model validation was performed using bootstraps with 1000 resampling's to quantify the overfitting of the modeling strategy and predict the future performance of the model. Harrell's C-statistic was calculated to assess the discrimination of the predictive models. A time-dependent receiver operating characteristic curve (t-ROC) analysis was carried out to calculate the area under the t-ROC (t-AUC). Besides, the interaction between treatment after the first TACE and 3-year predicted survival was graphed using a generalized additive model.

All analyses were completed with R 3.6.1 and Empower (www.empowerstats.com, X&Y solutions, Inc. Boston, MA). All statistical tests used in this study were two-sided, and the difference was considered statistically significant for P-values less than 0.05.

## 3. Results

### 3.1 Baseline characteristics of cohorts before first TACE

By the end of the follow-up at December 2016, 351 of 597 patients (58.8%) in the derivation cohort, and 394 of 920 patients in the validation cohort had died (42.8%). The median patient survival was 16.2 months (95% CI: 0.9-115.3) months for the derivation cohort and 19.2 months (0.9-98.5) for the validation cohort. The baseline characteristics of the study population are reported in Table 1 (also see Table S1).

### 3.2 Chemoembolization construction of the nomogram

**Figure 2:**
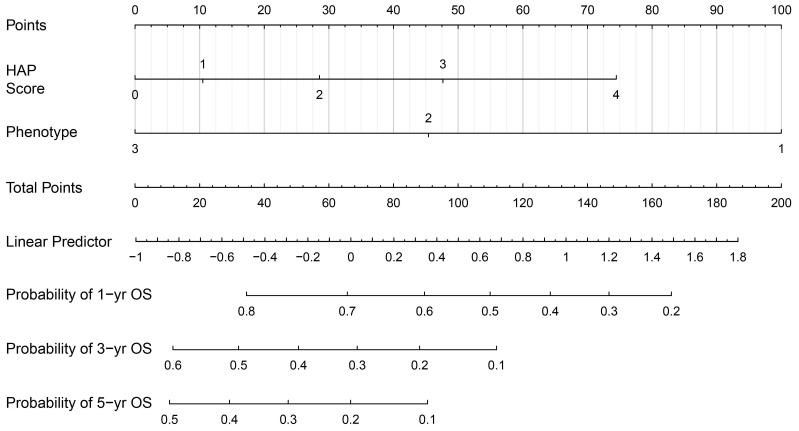
** The smHAP-II nomogram to predict the overall survival after first transarterial chemoembolization.** Logic(P)=0.15763*(HAP score=1) +0.42911*(HAP score=2) +0.71637*(HAP score=3) +1.11877* (HAP score=4) - 0.81905*(Subphenotype=2) -1.50174*(Subphenotype =3). HCC, hepatocellular carcinoma; TACE, transarterial chemoembolization; HAP, Hepatoma Arterial-embolization Prognostic.

Next, we established a new prognostic model, which we termed the 'smHAP- II' by incorporating the subphenotypes based on latent class analysis into the original HAP score. Each included patient had one individualized grade, which was defined by the sum of the points from HAP scores and phenotypes. The projections from total points (range, 0-200) shown on the scales indicated the predicted probabilities of 1-, 3- and 5-year OS (Figure 2).

### 3.3 Accuracy comparison between scores

**Figure 3:**
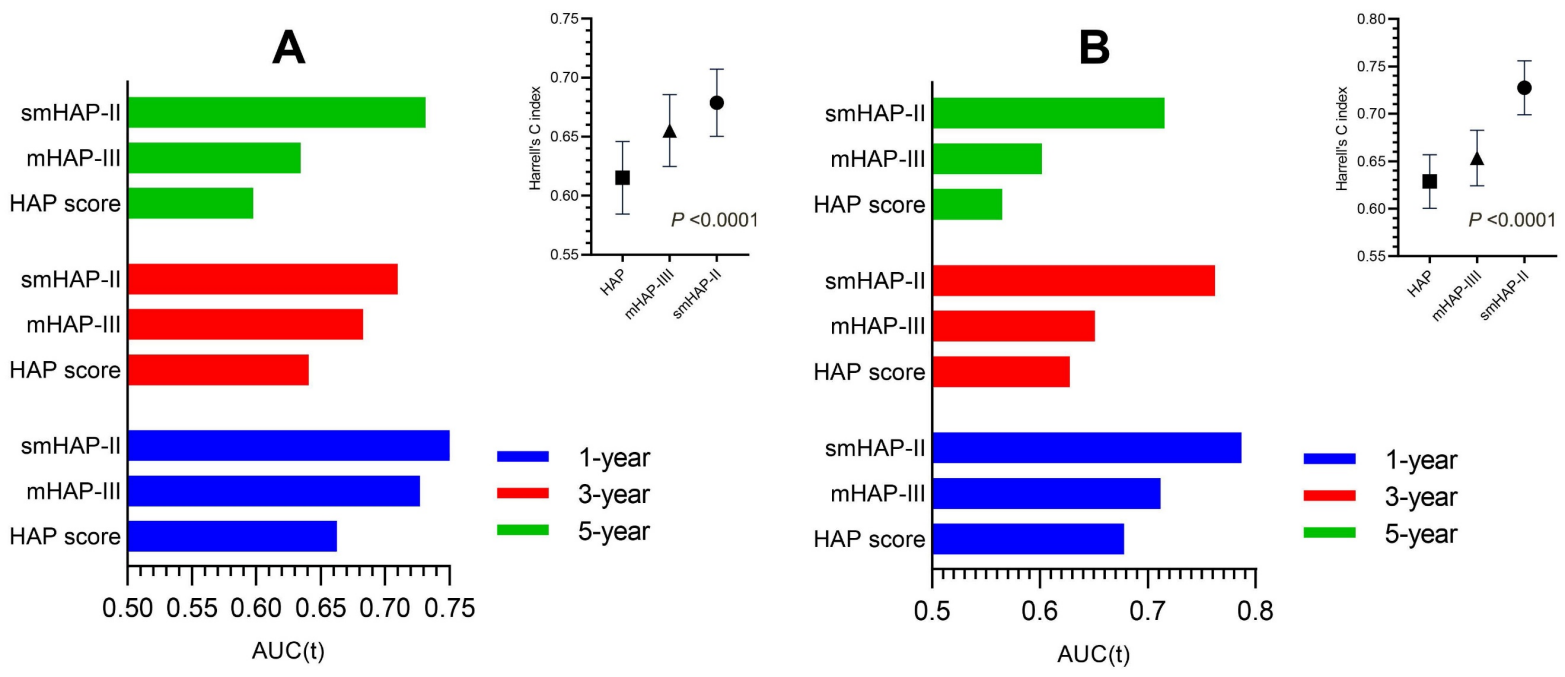
** Comparison of the performance and discriminative ability between the current model and other models.** (A) the derivation cohort; (B) the external cohort. Difference among three models was compared by Likelihood Ratio Test.

The Harrell's C-index of the nomogram was 0.679 (SD: 0.029) for the derivation cohort and 0.727(SD:0.029) for the external cohort, with a bootstrap-corrected C index of 0.679 for interval validation. In the derivation cohort, the t-AUCs to predict 1, 3, and 5-year OS were 0.750, 0.710, and 0.732 for the derivation cohort, while 0.789, 0.762, and 0.715 for the external cohort, indicating high discrimination of the model. To further evaluate the generalizability of model performance, we conducted the comparison for the HAP, mHAP-Ⅲ and our model in both cohorts. The accuracy of the smHAP-II model was superior to the HAP score and mHAP- III model in both cohorts (All *P* < 0.0001), which was shown in Figure 3.

### 3.4 Clinical application of the nomogram

**Figure 4:**
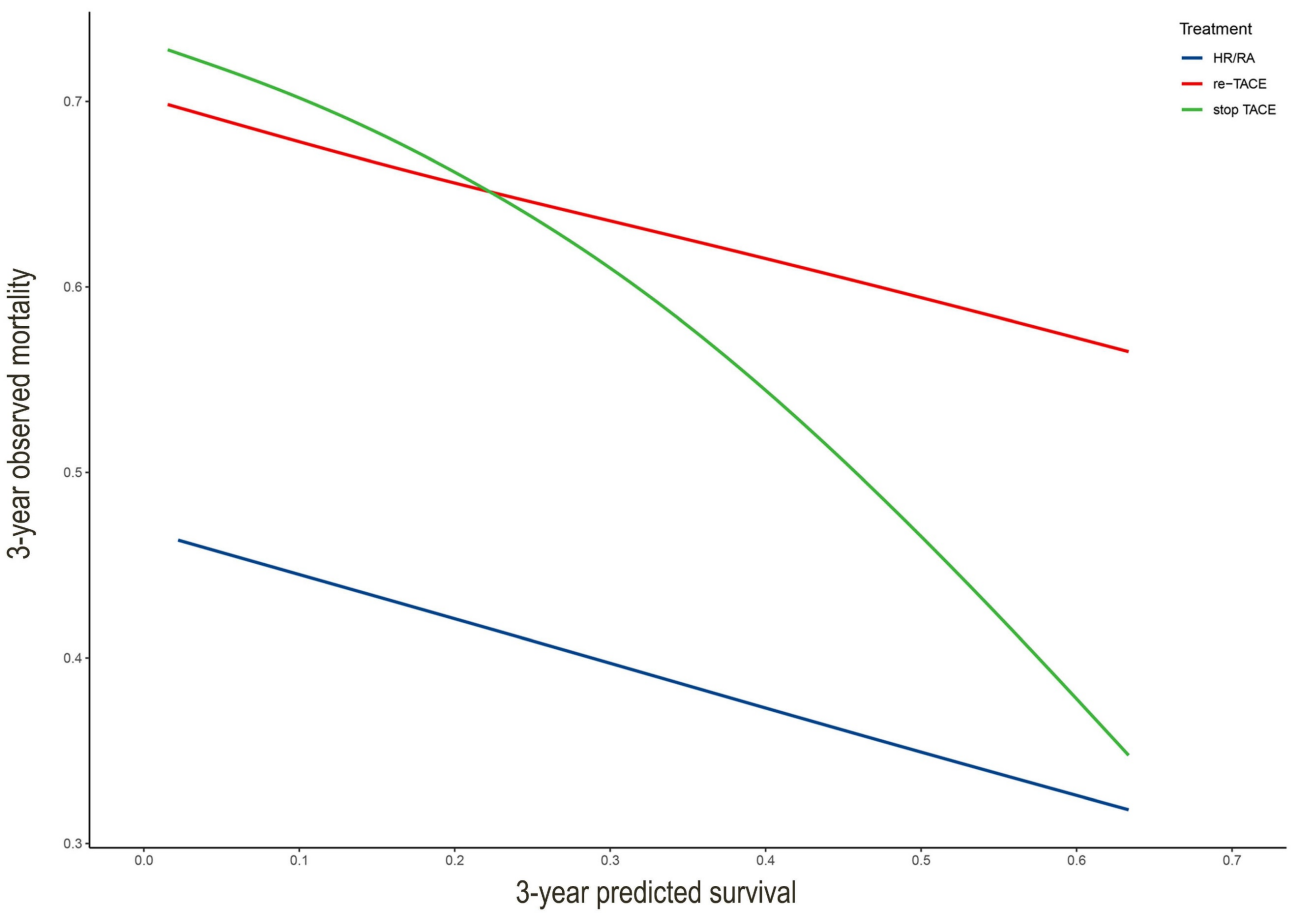
** The comparison with different treatments of the calibration plots of the nomogram at 3 years.** HA/RA: hepatic resection or radiofrequency or microwave ablation.

We drew the curve based on 3-year predicted survival and 3-year observed mortality with three treatments after the first TACE (Figure 4). It showed that whatever the 3-year predicted survival probability, the benefit for hepatic resection or radiofrequency or microwave ablation was greater than that of either the "re-TCAE" or "stop-TACE" scheme. Besides, the lines for re-TACE and stop-TACE cross at 0.23, indicating that patients with a 3-year predicted survival >23% would not benefit from TACE.

## 4. Discussion

In this large-scale multi-center cohort study, we developed and validated an individual smHAP-Ⅱ nomogram for uHCC patients after TACE. We found that adding postoperative subphenotypes could significantly enhanced the prognostic performance of the HAP score. Next, compared with the existing the HAP score and mHAP-Ⅲ score, our new model showed the best discrimination in the intermediate-stage HCC patients. Moreover, our study indicated that patients with a 3-year predicted survival >23% would not benefit from TACE.

Due to the heterogeneity of intermediate-stage HCC and the widespread application of TACE with the recommended guidelines, scores have been developed to predict survival after TACE 4, 5, 16, 22, 23, 25, 37. The HAP score as the first of these scores has been internationally validated without satisfactory accuracy 29. A new HAP score 24 (referred to as the modified HAP-II, mHAP-II) that incorporates tumor number has the advantage of easy application and simplicity but does not enable individual prognosis prediction. Therefore, Cappelli et al. developed a modified HAP-III system that included HAP variables along with tumor numbers in the continuous form 30.

Given that, a TACE-specific subphenotypes based on LCA analysis had been identified, which was strongly associated with clinical outcomes (Phenotype 1: P.S. score 1, stage progress, more intrahepatic lesions, and new intrahepatic lesions), TACE-responsive subphenotype (Phenotype 3: P.S. score 0, No intrahepatic lesions and new intrahepatic lesions), compared to TACE-intermediate subphenotype (Phenotype 2) 32. Our newly developed subphenotypes modified HAP-II aimed at evaluating survival and making treatment decisions with the least and most accessible characteristics. Meanwhile, our models stratify survival better than the currently available HAP and mHAP-III scores in C-index and AUC.

To date, it remains unknown whether TACE should be continued or abandoned after initial TACE for intermediate-stage HCC. The optimal number of sessions before switching treatment is also debatable. Our study demonstrated that hepatic resection or radiofrequency or microwave ablation might be the best choice of intermediate-stage HCC receiving TACE as first-line treatment based on 3-year predicted survival. This result ties well with previous studies that whenever possible, potentially curative treatments should be preferred to TACE repetition in case of non-response or at the time of cancer recurrence after the first TACE treatment since the survival of HCC patients is largely determined by the more effective treatment received, irrespective of the therapeutic sequence adopted 38, 39. We speculate that this might be due to the strict indication and contraindication 19 of these treatments. Therefore, it was not surprising that, after a first-line TACE, the adoption of treatment that can provide a higher survival benefit was associated with a better prognosis 38. Moreover, it has already been demonstrated that surgical treatment of HCC recurrence is a favorable prognostic factor 40-42. Therefore, the principle of first considering the therapy with the highest survival benefit is also valid in the second-line setting in case of non-response or recurrence after the frontline therapy 43. Although, we lacked information on molecularly targeted agents and immune checkpoint inhibitors due to the retrospective study. Systemic treatment includes molecularly targeted agents compared to repeated TACE is the recommended option for those BCLC-B patients whose first-line TACE is not feasible or fails 44. Compared with sorafenib, microwave ablation may be a more reasonable alternative treatment for intermediate-stage hepatocellular carcinoma (HCC) patients with tumor size > 7 cm and tumor number ≤ 5 after TACE refractoriness 45. Overall, our findings are in accordance with the findings reported. Though Chen S. et al. 46 recommend three conventional TACE sessions before switching to another treatment for nonresponding patients with intermediate-stage hepatocellular carcinoma, our results suggest that stop-TACE may be the better choice for to who would probably receive the greater chance of survival. The reason for this result may be that repeated TACE reduces the treatment effect and induces liver function impairment.

There are several limitations to our study. First of all, it was a retrospective study with inherent defects. Second, most of our patients were living in the south of China, which might limit the expansion of the nomogram. It should be noticed that with the development of systemic treatment, including molecularly targeted agents and immune checkpoint inhibitors, the retreatment effect following the first TACE was not completely assessed. However, this limitation was not affecting our new model. In addition, further research is necessary to validate our results with a large, multicenter patient cohort.

In conclusion, we developed robust LCA-based subphenotypes modified HAP- II score to predict the long-term OS of patients with intermediate-stage HCC after first-line TACE. It was validated that the nomogram compared with the HAP scores and mHAP-III scores, was associated with a good performance in predicting 1-, 3- and 5-year OS of the patients.

## Supplementary Material

Supplementary table.

## Figures and Tables

**Table 1:** Baseline characteristics of patients between derivation and external cohorts before first TACE treatment.

	Derivation Cohort	External Cohort	P-value
N	597	920	
Age(yr)	53.3 ± 12.2	52.5 ± 11.9	0.21
Gender			<0.001
Male	547 (91.6%)	427 (46.4%)	
Female	50 (8.4%)	493 (53.6%)	
ALB (g/L)	38.9 ± 5.6	38.9 ± 5.2	0.740
Log TBil (umol/L)	1.3 ± 0.2	1.3 ± 0.3	0.538
Child-Pugh class (missing data, n=20; 75)			0.397
A	508 (88.0%)	731 (86.5%)	
B	69 (12.0%)	114 (13.5%)	
AFP (ng/ml) (missing data, n=27; 58)			0.855
<200	265 (46.5%)	405 (47.0%)	
≥200	305 (53.5%)	457 (53.0%)	
Major tumor size (cm)	7.3 ± 3.7	7.0 ± 3.5	0.134
Location of Lesions			<0.001
Left lobe	14 (2.3%)	58 (6.3%)	
Right lobe	204 (34.2%)	333 (36.2%)	
Both lobe	379 (63.5%)	529 (57.5%)	
Intrahepatic lesion number			0.001
2	148 (24.8%)	310 (33.7%)	
3	47 (7.9%)	65 (7.1%)	
>3	402 (67.3%)	545 (59.2%)	

Differences are compared using the chi-square test (or Fisher's exact test) for categorical measures and Kruskal-Wallis test for continuous measures. Numbers that do not add up to 597 or 920 are attributable to missing data.

## Abbreviations

TACE: transarterial chemoembolization (TACE)
HCC: hepatocellular carcinoma
BCLC: Barcelona Clinic Liver Cancer
HAP: Hepatoma Arterial-embolization Prognostic
ALBI: albumin-bilirubin
ART: Assessment for Retreatment with TACE
ABCR: alpha-fetoprotein, BCLC, Child-Pugh, and response
SNACOR: tumour Size, tumour Number, baseline Alpha-foetoprotein level, Child-Pugh class and Objective radiological Response
LCA: latent class analysis
ROC: receiver operator characteristic

## References

[1] Forner A, Reig M, Bruix J (2018). Hepatocellular carcinoma. Lancet.

[2] Forner A, Llovet JM, Bruix J (2012). Hepatocellular carcinoma. Lancet.

[3] Sung H, Ferlay J, Siegel RL, Laversanne M, Soerjomataram I, Jemal A (2021). Global Cancer Statistics 2020: GLOBOCAN Estimates of Incidence and Mortality Worldwide for 36 Cancers in 185 Countries. CA Cancer J Clin.

[4] Liver (2018). EAftSot. EASL Clinical Practice Guidelines: Management of hepatocellular carcinoma. J Hepatol.

[5] Qiu G, Jin Z, Chen X, Huang J (2020). Interpretation of guidelines for the diagnosis and treatment of primary liver cancer (2019 edition) in China. Glob Health Med.

[6] Kwok PC, Lam TW, Chan SC, Chung CP, Wong WK, Chan MK (2000). A randomized clinical trial comparing autologous blood clot and gelfoam in transarterial chemoembolization for inoperable hepatocellular carcinoma. Journal of Hepatology.

[7] Ikeda M, Mitsunaga S, Shimizu S, Ohno I, Takahashi H, Okuyama H (2014). Efficacy of sorafenib in patients with hepatocellular carcinoma refractory to transcatheter arterial chemoembolization. J Gastroenterol.

[8] Kim YI, Park JW, Kwak HW, Kim BH, Lee JH, Lee IJ (2014). Long-term outcomes of second treatment after initial transarterial chemoembolization in patients with hepatocellular carcinoma. Liver Int.

[9] Kang JK, Kim MS, Cho CK, Yang KM, Yoo HJ, Kim JH (2012). Stereotactic body radiation therapy for inoperable hepatocellular carcinoma as a local salvage treatment after incomplete transarterial chemoembolization. Cancer.

[10] Hatooka M, Kawaoka T, Aikata H, Morio K, Kobayashi T, Hiramatsu A (2016). Comparison of Outcome of Hepatic Arterial Infusion Chemotherapy and Sorafenib in Patients with Hepatocellular Carcinoma Refractory to Transcatheter Arterial Chemoembolization. Anticancer Res.

[11] Kudo M, Finn RS, Qin S, Han KH, Ikeda K, Piscaglia F (2018). Lenvatinib versus sorafenib in first-line treatment of patients with unresectable hepatocellular carcinoma: a randomised phase 3 non-inferiority trial. Lancet.

[12] El-Khoueiry AB, Sangro B, Yau T, Crocenzi TS, Kudo M, Hsu C (2017). Nivolumab in patients with advanced hepatocellular carcinoma (CheckMate 040): an open-label, non-comparative, phase 1/2 dose escalation and expansion trial. Lancet.

[13] Bruix J, Qin S, Merle P, Granito A, Huang YH, Bodoky G (2017). Regorafenib for patients with hepatocellular carcinoma who progressed on sorafenib treatment (RESORCE): a randomised, double-blind, placebo-controlled, phase 3 trial. Lancet.

[14] Kelley RK, Verslype C, Cohn AL, Yang TS, Su WC, Burris H (2017). Cabozantinib in hepatocellular carcinoma: results of a phase 2 placebo-controlled randomized discontinuation study. Ann Oncol.

[15] European Association For The Study Of The L, European Organisation For R, Treatment Of C (2012). EASL-EORTC clinical practice guidelines: management of hepatocellular carcinoma. J Hepatol.

[16] Bolondi L, Burroughs A, Dufour JF, Galle PR, Mazzaferro V, Piscaglia F (2012). Heterogeneity of patients with intermediate (BCLC B) Hepatocellular Carcinoma: proposal for a subclassification to facilitate treatment decisions. Seminars in liver disease.

[17] Ha Y, Shim JH, Kim SO, Kim KM, Lim YS, Lee HC (2014). Clinical appraisal of the recently proposed Barcelona Clinic Liver Cancer stage B subclassification by survival analysis. J Gastroenterol Hepatol.

[18] Sangro B, Salem R (2014). Transarterial chemoembolization and radioembolization. Seminars in liver disease.

[19] Mazzaferro V, Llovet JM, Miceli R, Bhoori S, Schiavo M, Mariani L (2009). Predicting survival after liver transplantation in patients with hepatocellular carcinoma beyond the Milan criteria: a retrospective, exploratory analysis. Lancet Oncol.

[20] Kadalayil L, Benini R, Pallan L, O'Beirne J, Marelli L, Yu D (2013). A simple prognostic scoring system for patients receiving transarterial embolisation for hepatocellular cancer. Ann Oncol.

[21] Yamakado K, Miyayama S, Hirota S, Mizunuma K, Nakamura K, Inaba Y (2014). Subgrouping of intermediate-stage (BCLC stage B) hepatocellular carcinoma based on tumor number and size and Child-Pugh grade correlated with prognosis after transarterial chemoembolization. Japanese journal of radiology.

[22] Yousuf F, Cross TJ, Palmer D (2014). The ART strategy: sequential assessment of the ART score predicts outcome of patients with hepatocellular carcinoma re-treated with TACE. J Hepatol.

[23] Adhoute X, Penaranda G, Naude S, Raoul JL, Perrier H, Bayle O (2015). Retreatment with TACE: the ABCR SCORE, an aid to the decision-making process. J Hepatol.

[24] Kim BK, Shim JH, Kim SU, Park JY, Kim DY, Ahn SH (2016). Risk prediction for patients with hepatocellular carcinoma undergoing chemoembolization: development of a prediction model. Liver Int.

[25] Kim JH, Shim JH, Lee HC, Sung KB, Ko HK, Ko GY (2017). New intermediate-stage subclassification for patients with hepatocellular carcinoma treated with transarterial chemoembolization. Liver Int.

[26] Pinato DJ, Sharma R, Allara E, Yen C, Arizumi T, Kubota K (2017). The ALBI grade provides objective hepatic reserve estimation across each BCLC stage of hepatocellular carcinoma. J Hepatol.

[27] Wang Q, Xia D, Bai W, Wang E, Sun J, Huang M (2019). Development of a prognostic score for recommended TACE candidates with hepatocellular carcinoma: A multicentre observational study. J Hepatol.

[28] Hucke F, Pinter M, Graziadei I, Bota S, Vogel W, Muller C (2014). How to STATE suitability and START transarterial chemoembolization in patients with intermediate stage hepatocellular carcinoma. J Hepatol.

[29] Pinato DJ, Arizumi T, Allara E, Jang JW, Smirne C, Kim YW (2015). Validation of the hepatoma arterial embolization prognostic score in European and Asian populations and proposed modification. Clin Gastroenterol Hepatol.

[30] Cappelli A, Cucchetti A, Cabibbo G, Mosconi C, Maida M, Attardo S (2016). Refining prognosis after trans-arterial chemo-embolization for hepatocellular carcinoma. Liver International: Official Journal of the International Association For the Study of the Liver.

[31] Han G, Berhane S, Toyoda H, Bettinger D, Elshaarawy O, Chan AWH (2020). Prediction of Survival Among Patients Receiving Transarterial Chemoembolization for Hepatocellular Carcinoma: A Response-Based Approach. Hepatology.

[32] Chen S, Guo A, Lu L, Lin S, Hu X, Zhu L (2022). Latent Class Analysis of Subphenotypes in Intermediate-Stage Hepatocellular Carcinoma after Transarterial Chemoembolization. J Cancer.

[33] Lu L, Su Z, Zheng P, Wu Z, Zhang Y, He H (2020). Association between platelet count and hepatocellular carcinoma overall survival: a large retrospective cohort study. BMJ open.

[34] Lu L, Zhang Y, Zheng P, Wu Z, Wang X, Chen Y (2020). Elevated Platelet Count is Associated with Poor Survival After Transarterial Chemoembolization Treatment in Patients with Hepatocellular Carcinoma: A Cohort Study. Journal of hepatocellular carcinoma.

[35] Shen L, Qi Z, Pi G, Huang J, Li C, Pan T (2018). Dynamically prognosticating patients with hepatocellular carcinoma through survival paths mapping based on time-series data. Nature Publishing Group.

[36] Collins GS, Reitsma JB, Altman DG, Moons KG (2015). Transparent reporting of a multivariable prediction model for individual prognosis or diagnosis (TRIPOD): the TRIPOD Statement. BMC Med.

[37] Zhou J, Sun HC, Wang Z, Cong WM, Wang JH, Zeng MS (2018). Guidelines for Diagnosis and Treatment of Primary Liver Cancer in China (2017 Edition). Liver Cancer.

[38] Pelizzaro F, Haxhi S, Penzo B, Vitale A, Giannini EG, Sansone V (2022). Transarterial Chemoembolization for Hepatocellular Carcinoma in Clinical Practice: Temporal Trends and Survival Outcomes of an Iterative Treatment. Front Oncol.

[39] Vitale A, Farinati F, Pawlik TM, Frigo AC, Giannini EG, Napoli L (2019). The concept of therapeutic hierarchy for patients with hepatocellular carcinoma: A multicenter cohort study. Liver Int.

[40] Erridge S, Pucher PH, Markar SR, Malietzis G, Athanasiou T, Darzi A (2017). Meta-analysis of determinants of survival following treatment of recurrent hepatocellular carcinoma. Br J Surg.

[41] Tabrizian P, Jibara G, Shrager B, Schwartz M, Roayaie S (2015). Recurrence of hepatocellular cancer after resection: patterns, treatments, and prognosis. Ann Surg.

[42] Vitale A, Farinati F, Noaro G, Burra P, Pawlik TM, Bucci L (2018). Restaging Patients With Hepatocellular Carcinoma Before Additional Treatment Decisions: A Multicenter Cohort Study. Hepatology.

[43] Vitale A, Trevisani F, Farinati F, Cillo U (2020). Treatment of Hepatocellular Carcinoma in the Precision Medicine Era: From Treatment Stage Migration to Therapeutic Hierarchy. Hepatology.

[44] Reig M, Forner A, Rimola J, Ferrer-Fàbrega J, Burrel M, Garcia-Criado Á (2022). BCLC strategy for prognosis prediction and treatment recommendation: The 2022 update. Journal of Hepatology.

[45] Chen S, Shi M, Shen L, Qi H, Wan W, Cao F (2020). Microwave ablation versus sorafenib for intermediate-Stage Hepatocellular carcinoma with transcatheter arterial chemoembolization refractoriness: a propensity score matching analysis. International Journal of Hyperthermia.

[46] Chen S, Peng Z, Zhang Y, Chen M, Li J, Guo R (2021). Lack of Response to Transarterial Chemoembolization for Intermediate-Stage Hepatocellular Carcinoma: Abandon or Repeat?. Radiology.

