# Supplementary material for: Postoperative subphenotypes modified the hepatoma arterial-embolization prognostic score: A novel smHAP-II nomogram
Source: J Cancer. 2024 Mar 25;15(10):2940–7. doi: 10.7150/jca.91175 (PMC11064269; doi:10.7150/jca.91175)
Supplement: Supplementary file 1 — Supplementary table. [file jcav15p2940s1.pdf]

Table S1. Comparison of key clinical data points between derivation and external cohorts after first TACE treatment.

	Derivation cohort (n=597)	External cohort (n=920)	*P*-value
Age（yr）			0.015
<55	283 (47.4%)	495 (53.8%)	
≥55	314 (52.6%)	425 (46.2%)	
Gender			<0.001
male	547 (91.6%)	427 (46.4%)	
female	50 (8.4%)	493 (53.6%)	
PS score			0.588
0	490 (82.1%)	765 (83.2%)	
1	107 (17.9%)	155 (16.8%)	
Child-Pugh class			0.362
A	53 (17.1%)	62 (15.6%)	
B	253 (81.6%)	324 (81.6%)	
C	4 (1.3%)	11 (2.8%)	
No. of intrahepatic lesions			<0.001
0	159 (26.6%)	354 (38.5%)	
<3	155 (26.0%)	217 (23.6%)	
≥3	283 (47.4%)	349 (37.9%)	
Diameter of main tumor（cm）			<0.001
0	159 (26.6%)	358 (38.9%)	
<5	155 (26.0%)	234 (25.4%)	
≥5	283 (47.4%)	328 (35.7%)	
Location of lesions			<0.001
none	159 (26.6%)	355 (38.6%)	
left/right	163 (27.3%)	237 (25.8%)	
both	275 (46.1%)	328 (35.7%)	
New intrahepatic lesions			0.964
no	506 (84.8%)	779 (84.7%)	
yes	91 (15.2%)	141 (15.3%)	
Vascular invasion			0.916
no	552 (92.5%)	852 (92.6%)	
yes	45 (7.5%)	68 (7.4%)	
Distant metastasis			0.903
no	544 (91.1%)	840 (91.3%)	
yes	53 (8.9%)	80 (8.7%)	
Lymph node metastasis			0.897
no	561 (94.0%)	866 (94.1%)	
yes	36 (6.0%)	54 (5.9%)	

Differences are compared using the chi-square test (or Fisher's exact test) for categorical measures and Kruskal−Wallis test for continuous measures. Numbers that do not add up to 597 or 920 are attributable to missing data.
